# Early SGLT2 Inhibitor Therapy in Acute Coronary Syndrome: Mitigating Adverse Remodeling in High-Risk Phenotypes—A Real-World Study

**DOI:** 10.3390/medicina62010205

**Published:** 2026-01-19

**Authors:** Teodora Mateoc, Ioana-Maria Suciu, Dan Gaiță, Andor Minodora, Roxana Popescu, Tania Vlad, Corina Flangea, Călin Muntean, Daliborca-Cristina Vlad

**Affiliations:** 1 Doctoral School, Faculty of Medicine, “Victor Babeș” University of Medicine and Pharmacy, 2nd Eftimie Murgu Square, 300041 Timisoara, Romania; teodora.mateoc@umft.ro (T.M.); vlad.daliborca@umft.ro (D.-C.V.); 2 Institute of Cardiovascular Diseases Timișoara, 13A Gheorghe Adam Street, 300310 Timisoara, Romania; dgaita@cardiologie.ro; 3 Multidisciplinary Heart Research Center, “Victor Babeș” University of Medicine and Pharmacy, 300041 Timisoara, Romania; andor.minodora@umft.ro; 4 Medical Semiology II Discipline, Internal Medicine Department, “Victor Babeș” University of Medicine and Pharmacy, 2nd Eftimie Murgu Square, 300041 Timisoara, Romania; 5 Department of Biochemistry and Pharmacology, Faculty of Medicine, “Victor Babeș” University of Medicine and Pharmacy, 2nd Eftimie Murgu Square, 300041 Timisoara, Romania; popescu.roxana@umft.ro (R.P.); tania.vlad@umft.ro (T.V.); flangea.corina@umft.ro (C.F.); 6 Department III-Functional Sciences, Medical Informatics and Biostatistics, “Victor Babeș” University of Medicine and Pharmacy, 2nd Eftimie Murgu Square, 300041 Timisoara, Romania; cmuntean@umft.ro

**Keywords:** SGLT2 inhibitors, acute coronary syndrome, left ventricular remodeling, real-world evidence, echocardiography, SGLT2 inhibitors, ventricular remodeling, multivariable regression

## Abstract

*Background and Objectives:* SGLT2 inhibitors are foundational in heart failure therapy, yet their impact on left ventricular (LV) remodeling immediately following acute coronary syndrome (ACS) remains less defined. This study evaluated the association between early SGLT2 inhibitor initiation and structural recovery in a real-world post-ACS cohort. *Materials and Methods:* We conducted a retrospective observational study including 238 revascularized ACS patients, stratified into an SGLT2 inhibitor group (n = 71) and a control group (n = 167). Changes in LV ejection fraction (LVEF) and indexed LV mass (LVMi) were assessed by echocardiography at baseline and follow-up (mean 286 days). Multivariable regression models were adjusted for baseline imbalances and tested for interactions with diabetes status. *Results:* A significant “confounding by indication” was observed; the SGLT2 group presented a high-risk phenotype with higher diabetes prevalence (56.3% vs. 25.7%, *p* < 0.001), lower baseline LVEF (38.3% vs. 43.3%), and greater hypertrophy. After adjustment, statistical independence was attenuated by baseline severity, yet the SGLT2 group achieved follow-up structural outcomes comparable to lower-risk controls. Interaction analysis indicated these trends were consistent regardless of diabetes status (*p* > 0.05). *Conclusions:* In this high-risk ACS population, early SGLT2 inhibitor therapy was associated with stabilization of cardiac structure. Despite a profound baseline disadvantage, the recovery trajectory effectively aligned with that of a lower-risk population, highlighting a clinically relevant pattern of structural stabilization consistent across metabolic subgroups.

## 1. Introduction

Acute myocardial infarction (AMI) remains a preeminent cause of cardiovascular morbidity and mortality globally [1], despite the widespread implementation of early reperfusion strategies and secondary prevention pharmacotherapy [2,3,4]. The survival of the acute ischemic event is frequently followed by a pervasive and maladaptive process known as adverse left ventricular (LV) remodeling [5]. This complex phenomenon involves genomic, molecular, and cellular alterations that manifest macroscopically as progressive ventricular dilatation, distortion of chamber geometry, and myocardial hypertrophy [6,7]. The pathophysiology of post-infarction remodeling is multiphasic [8,9], transitioning [10] from an initial phase of necrotic resorption and infarct expansion to a chronic phase driven by neurohormonal activation and persistent inflammation, which promote interstitial fibrosis and cardiomyocyte hypertrophy in the non-infarcted remote myocardium [11,12]. It is this structural deviation that serves as the primary substrate for the development of heart failure (HF) [13,14,15], malignant arrhythmias, and adverse long-term cardiovascular outcomes [16]. Consequently, the attenuation or reversal of this process—termed “reverse remodeling”—has become a surrogate endpoint of therapeutic efficacy in modern cardiology [17,18,19].

Sodium-glucose cotransporter-2 inhibitors (SGLT2i) have undergone a remarkable evolution from their inception as glucose-lowering agents for type 2 diabetes mellitus to their current status as a foundational pillar of heart failure therapy [20,21]. Landmark randomized controlled trials (RCTs), including DAPA-HF [22], EMPEROR-Reduced [23], and EMPEROR-Preserved [24], have unequivocally demonstrated that agents such as dapagliflozin and empagliflozin significantly reduce the risk of heart failure hospitalization and cardiovascular death across the entire spectrum of left ventricular ejection fractions, largely independent of glycemic status [22,23,24]. The mechanisms underpinning these benefits are pleiotropic, extending beyond glycosuria to include direct cardioprotective effects through the modulation of myocardial energetics, suppression of inflammation, reduction of oxidative stress, and inhibition of the sodium-hydrogen exchanger 1 (NHE1) [25,26].

Despite the robust evidence in chronic heart failure, the role of SGLT2 inhibitors in the immediate aftermath of Acute Coronary Syndrome (ACS) remains an area of active investigation. While recent guidelines have endorsed the early initiation of these agents, the evidence base specifically addressing structural endpoints in the post-ACS population is less definitive [27]. Recent trials such as EMPACT-MI [28] and DAPA-MI [29] have confirmed the safety and suggested benefits in reducing heart failure hospitalizations, but data regarding granular, longitudinal changes in cardiac structure remain limited, particularly in unselected, “real-world” populations [28,30]. Timely initiation, within the early post-ACS phase, may help prevent maladaptive ventricular remodeling before structural changes become established, thereby preserving cardiac function and long-term outcomes [20,31,32]. In this context, the present single-center retrospective observational cohort study was designed to rigorously evaluate longitudinal echocardiographic remodeling patterns in patients admitted with ACS and treated with percutaneous coronary intervention (PCI). The primary objective was to determine whether exposure to SGLT2 inhibitor therapy in the early post-infarction period is independently associated with favorable changes in left ventricular systolic function and structure. By utilizing detailed multivariable regression models to account for baseline structural and metabolic heterogeneity, this study aims to clarify the “phenotype-dependent” nature of cardiac remodeling and the independent contribution of SGLT2 inhibition to structural recovery in a contemporary, real-world setting.

## 2. Materials and Methods

### 2.1. Study Design and Ethical Compliance

This investigation was conducted as a single-center retrospective observational cohort study at the Institute of Cardiovascular Disease Timișoara, Western Romania. The study protocol was developed in strict adherence to the STROBE (Strengthening the Reporting of Observational Studies in Epidemiology) guidelines, ensuring comprehensive reporting of study design, participant selection, variable definition, and statistical methods [33]. The study incorporated data spanning the period from January 2023 to June 2024. Patient consent was waived due to the retrospective nature of the study. Ethical approval was obtained from the institutional review board (protocol code 5301, date of approval 9 July 2024), and the study was conducted in accordance with the ethical principles stated in the Declaration of Helsinki.

### 2.2. Patient Population and Eligibility Criteria

The source population comprised consecutive adult patients admitted to the coronary care unit with a confirmed diagnosis of acute coronary syndrome (ACS). Inclusion criteria were as follows: (1) confirmed ACS, classified as either ST-segment elevation myocardial infarction (STEMI) or non-ST-segment elevation acute coronary syndrome (NSTE-ACS); (2) successful revascularization via percutaneous coronary intervention (PCI) during the index hospitalization; and (3) availability of a complete transthoracic echocardiogram (TTE) at baseline and at least one follow-up TTE between 3 and 34 months post-discharge. Exclusion criteria included the following: incomplete echocardiographic or laboratory data, end-stage renal disease (eGFR < 20 mL/min/1.73 m^2^) or dialysis dependency, active malignancy, severe anemia, sepsis, or severe valvular heart disease. An initial screening of 382 patients identified 238 patients meeting all criteria, who constituted the final analytic cohort. Patients were stratified into two groups: the SGLT2 group (n = 71) and the non-SGLT2 group (n = 167).

### 2.3. Study Outcomes

The primary outcomes were longitudinal changes (Δ) in echocardiographic parameters of left ventricular structure and function: (1) change in left ventricular ejection fraction (ΔLVEF); (2) change in left ventricular mass (ΔLVM) and indexed LVM (ΔLVMi); and (3) change in relative wall thickness (ΔRWT). Secondary outcomes included descriptive analysis of rehospitalization causes during follow-up.

### 2.4. Echocardiographic Assessment

Transthoracic echocardiography was performed using a Vivid S5 (GE Healthcare, Chicago, IL, USA) system. Key parameters included: Left Ventricular Ejection Fraction (LVEF) calculated using the biplane Simpson method; Left Ventricular Mass (LVM) estimated using the linear method (Devereux formula); Indexed LVM (LVMi) normalized to body surface area (BSA); Relative Wall Thickness (RWT) calculated as (2 × PW)/LVEDD; and Longitudinal Change (Δ) calculated as the baseline value minus the follow-up value.

### 2.5. Statistical Analysis

Continuous variables were assessed for normality using the Shapiro–Wilk test. Normally distributed data are presented as mean ± standard deviation (SD), while non-normally distributed data are presented as median with interquartile range (IQR). Categorical variables are expressed as counts and percentages. Baseline characteristics were compared using Student’s *t*-test or Mann–Whitney U test, and the Chi-square (χ^2^) test or Fisher’s exact test. Multivariable linear regression models were constructed for each dependent variable (ΔLVEF, ΔLVM, ΔLVMi, ΔRWT), adjusted for baseline values of the outcome variable and critically, for the time interval between baseline and follow-up echocardiography to account for the heterogeneous follow-up durations inherent to real-world clinical practice. A two-sided *p*-value < 0.05 was considered statistically significant. Statistical analyses were performed using Orange: Data Mining Toolbox version 3.37.0 in Python (developed by the Bioinformatics Laboratory, Faculty of Computer and Information Science, University of Ljubljana, Ljubljana, Slovenia) and MedCalc Statistical Software version 22.015 (MedCalc Software Ltd., Ostend, Belgium). To formally assess whether the effect of SGLT2 inhibitor therapy on cardiac remodeling was modified by diabetes status, as suggested by reviewer feedback, we introduced interaction terms (SGLT2i × Diabetes) into the multivariable linear regression models. These models were constructed for each primary outcome (ΔLVEF, ΔLVMi, ΔLVM, and ΔRWT) and were similarly adjusted for the baseline value of the outcome variable and the time to follow-up. Acknowledging the significant baseline imbalances inherent to this observational study (“confounding by indication”), we considered alternative statistical approaches, including propensity score matching (PSM). A preliminary feasibility analysis revealed that while PSM could balance the measured covariates, it would come at the cost of a substantial reduction in sample size (an estimated loss of 15.5% of the SGLT2i group) and statistical power (a decrease from 83% to 62% for detecting a clinically meaningful 3% change in LVEF). Given that a primary goal was to analyze a “real-world” cohort, we prioritized the preservation of the full sample and statistical power. Therefore, multivariable regression was chosen as the primary method for adjustment, as it allows for the control of multiple confounders simultaneously while retaining the entire cohort, a common and accepted approach in large observational studies like EMPACT-MI and DAPA-MI.

## 3. Results

### 3.1. Baseline Clinical Characteristics and Confounding by Indication

**Figure 1:**
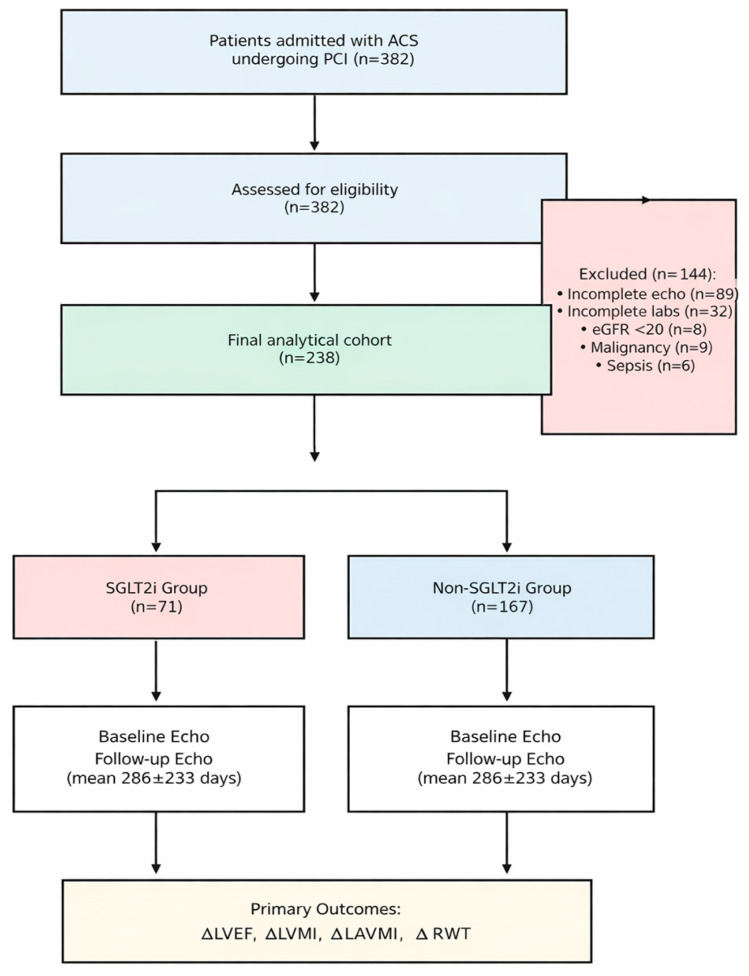
Study flow diagram depicting patient selection and group allocation.

The final analysis included 238 patients (Figure 1). The demographic and clinical profile of the cohort reflects a typical high-risk ACS population: predominantly male (76.1%), middle-aged (mean 60.3 ± 10.7 years), with a very high burden of classic cardiovascular risk factors. Hypertension was present in 86.1% of patients, and dyslipidemia in 98.7%. A stark imbalance was observed between the treatment groups at baseline, providing clear evidence of “confounding by indication.” Patients in the SGLT2 group were significantly more likely to have diabetes mellitus (56.3% vs. 25.7%, *p* < 0.001), reflecting the guideline-driven prioritization of these agents for diabetic patients with cardiovascular disease. The mean time to follow-up echocardiography was similar between groups (290.8 ± 208.1 days vs. 284.5 ± 243.8 days, *p* = 0.848), ensuring comparability of the remodeling phase captured (Table 1).

### 3.2. Baseline Echocardiographic Phenotype

**Figure 2:**
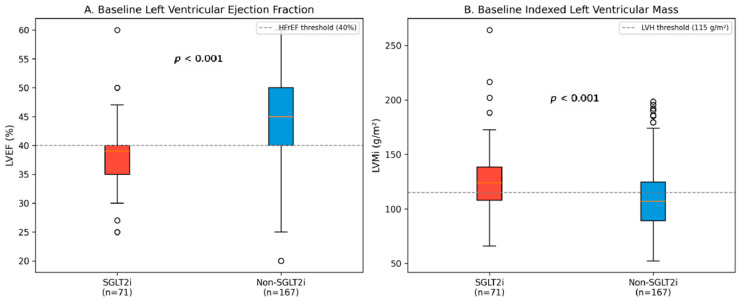
Baseline echocardiographic parameters comparison. (**A**) Baseline LVEF showing significantly lower values in the SGLT2i group; the dashed line represents the 40% threshold for heart failure with reduced ejection fraction (HFrEF). (**B**) Baseline LVMi demonstrating significantly higher values in the SGLT2i group; the dashed line represents the 115 g/m^2^; threshold for left ventricular hypertrophy (LVH).

The “confounding by indication” extended prominently to the structural phenotype of the heart. The SGLT2 group presented with a distinct “high-risk” structural phenotype: significantly lower systolic function (LVEF 38.3% vs. 43.3%, *p* < 0.001), and markedly increased myocardial mass (LVMi 125.8 vs. 110.3 g/m^2^, *p* < 0.001). This indicates that clinicians were appropriately targeting SGLT2 inhibitors toward patients with HFrEF and adverse structural remodeling, consistent with ESC guidelines (Table 2, Figure 2).

### 3.3. Longitudinal Remodeling Outcomes

**Figure 3:**
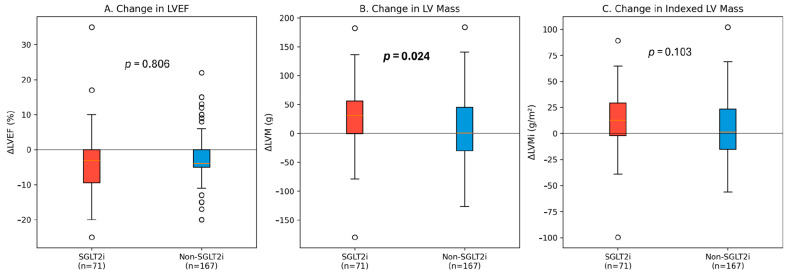
Longitudinal changes in echocardiographic parameters. Box plots showing the distribution of changes (Δ) in (**A**) LVEF, (**B**) LVM, and (**C**) LVMi between baseline and follow-up. The horizontal line at y = 0 represents no change from baseline.

Follow-up echocardiography was performed at a mean of 286 ± 233 days. Both groups showed evidence of recovery, but the patterns differed. The absolute change in LVEF (ΔLVEF) was not statistically different between groups (−3.30% vs. −3.04%, *p* = 0.806). However, the unadjusted reduction in LV mass was significantly greater in the SGLT2 group (ΔLVM 27.8 ± 53.8 g vs. 10.0 ± 55.5 g, *p* = 0.024) (Figure 3).

### 3.4. Adjusted Analysis: The Role of Baseline Heterogeneity

While unadjusted data suggested that SGLT2 inhibitors powerfully reduced LV mass, multivariable linear regression revealed that when models were adjusted for the baseline echocardiographic values and time to follow-up, the independent association between SGLT2 inhibitor therapy and structural improvement was attenuated and lost statistical significance. The primary determinant of follow-up LVEF and LVM was the baseline value of that parameter (Table 3).

### 3.5. Interaction Analysis by Diabetes Status

To address the hypothesis that the therapeutic impact of SGLT2 inhibitors might differ between diabetic and non-diabetic patients, we formally tested for statistical interaction. In multivariable models adjusted for baseline characteristics and follow-up duration, the interaction term (SGLT2i × Diabetes) was not statistically significant for any of the primary structural outcomes, including change in LVEF (*p* for interaction = 0.810), LVMi (*p* for interaction = 0.811), or LVM (*p* for interaction = 0.914). These findings suggest that, within the statistical power of this study, the association of SGLT2 inhibitor therapy with cardiac remodeling was consistent across patients regardless of their diabetes status. The detailed results of these interaction models are presented in Table 4.

## 4. Discussion

This temporal and clinical context contributed to marked baseline imbalances, with the SGLT2 group displaying a significantly higher burden of cardiometabolic comorbidities, particularly diabetes mellitus (56.3% vs. 25.7%, *p* < 0.001) and chronic kidney disease (14.1% vs. 6.0%). In the natural course of post-infarction recovery, such a high-risk phenotype is typically associated with accelerated adverse remodeling compared to patients without metabolic derangements. Consequently, the observation that SGLT2-treated patients achieved follow-up structural and functional outcomes comparable to the ‘healthier’ non-SGLT2 cohort—after multivariable adjustment—suggests a favorable clinical association regarding risk status. Rather than merely implying a lack of statistical independence, these results indicate that the remodeling trajectory of these high-risk patients closely paralleled that of the lower-risk population.

The central insight from this study is that cardiac remodeling in the real-world post-ACS setting is highly phenotype dependent. Our findings resonate with emerging concepts in the heart failure literature, which suggest that the efficacy of neurohormonal and metabolic modulators [34,35,36] is contingent upon the underlying structural and metabolic substrate of the myocardium. The SGLT2 group in our cohort represented distinct hypertrophic-ischemic phenotype—patients with concomitant diabetes, significant hypertrophy (high LVMi), and depressed systolic function. The unadjusted analysis showed massive mass regression in this group. While statistical adjustment attributed this primarily to the baseline mass itself, biological plausibility for a drug effect remains. Hypertrophied hearts are metabolically inefficient and energy-starved [37,38,39,40]. SGLT2 inhibitors promote the utilization of ketone bodies, a “super-fuel” that yields more ATP per molecule of oxygen consumed than fatty acids [41,42,43]. In the peri-infarct zone, where oxygen is scarce, this metabolic shift could theoretically alleviate energetic stress and facilitate the regression of hypertrophy [44,45,46,47]. Furthermore, emerging evidence suggests that the benefits of SGLT2 inhibitors extend beyond metabolic modulation to include pleiotropic effects that stabilize cardiac electrophysiology and improve structural integrity [48,49]. Our interaction analysis did not reveal a statistically significant difference in the association between SGLT2i therapy and cardiac remodeling in diabetic versus non-diabetic patients. While our study was likely underpowered to definitively rule out such interaction, these results suggest that the observed neutral association with structural changes was consistent across these subgroups.

Furthermore, recent sub-analyses from the EMPA-HEART CardioLink-6 trial [12] have shown that SGLT2 inhibitor-mediated regression of LV mass is most profound in patients with the highest baseline LVMi. Our data mirrors this: the group with high baseline mass (SGLT2 group) regressed the most. Whether this is purely statistical (regression to the mean) or biological (drug works best where substrate is worst) is a nuanced debate. Our regression analysis favors statistical explanation, but the biological signal cannot be entirely dismissed given the consistent pattern across studies.

The lack of an independent SGLT2 signal in our adjusted models aligns with the mixed results of recent post-MI trials like EMPACT-MI and DAPA-MI [28,29]. Unlike the chronic heart failure trials (DAPA-HF [22], EMPEROR [24]), which showed clear mortality benefits, these acute post-MI trials showed reductions in heart failure hospitalizations [48] but failed to reduce mortality significantly. This suggests that the immediate post-infarction phase—dominated by necrotic resorption, acute inflammation, and fluctuating loading conditions—may be a noisier environment where the subtle, long-term structural benefits of SGLT2 inhibitors are harder to isolate or take longer to manifest [50].

Importantly, the interpretation of our “null” adjusted findings warrants careful consideration. The absence of a statistically significant independent association should not be confounded with therapeutic futility. Given the profound baseline disadvantage of the SGLT2 group—characterized by significantly higher diabetes prevalence, worse renal function, lower ejection fraction, and greater myocardial hypertrophy, the fact that their structural outcomes converged with those of the metabolically healthier control group represents a clinically meaningful observation.

In essence, the use of SGLT2 inhibitors was associated with an absence of the accelerated adverse remodeling typically expected in such a high-risk cohort, with outcomes effectively converging with those of lower-risk patients. This observational pattern, while not demonstrating superiority, suggests a stabilization of cardiac structure that merits recognition.

### Limitations

Several limitations must be acknowledged. First, the retrospective nature of the study introduces inherent selection bias (“confounding by indication”), as evidenced by the significant baseline differences between groups. While we adjusted for this statistically, including the time interval between echocardiographic assessments, residual confounding is possible. Second, the sample size (n = 71 in the treated group) may have been underpowered to detect small but clinically meaningful differences in echocardiographic parameters. Third, our structural assessment was based on standard 2D echocardiography. While LVEF and LVM are robust and clinically validated endpoints, the absence of more sensitive imaging techniques, such as speckle-tracking echocardiography for global longitudinal strain (GLS) or cardiac magnetic resonance (CMR), is a notable limitation. These modalities are more adept at detecting subtle, early-stage changes in myocardial function and tissue composition (e.g., fibrosis, early dysfunction, regional strain abnormalities, fibrosis, scar vs. viable myocardium, higher risk of arrythmias and mortality). It is plausible that the neutral findings in our adjusted analyses may be due, in part, to the limited sensitivity of conventional echocardiography in detecting modest, albeit potentially important, therapeutic associations. A significant limitation, noted by our reviewers, is the wide variability in the follow-up period (3–34 months). This heterogeneity reflects the challenges of longitudinal data collection in a real-world setting, where patient adherence to scheduled appointments can vary. While we statistically controlled for follow-up duration in all our multivariable models to mitigate its impact as a confounder, we cannot exclude the possibility that this variability introduced noise into the measurement of remodeling, potentially obscuring subtle structural changes. Our analysis confirmed that follow-up duration was not significantly different between the treatment groups and did not correlate with the primary outcomes, providing some reassurance that it did not introduce a systematic bias. Post hoc power calculation was not performed due to the retrospective fixed sample size, but we acknowledge that the study may be underpowered to detect subtle changes in LVEF independent of baseline variables. Fourth, our primary statistical approach relied on multivariable regression rather than propensity score matching. This decision was made deliberately to preserve statistical power and maintain the full “real-world” cohort, but we acknowledge that residual, unmeasured confounding may still be present.

Finally, we relied on standard 2D echocardiographic parameters; more advanced modalities like Global Longitudinal Strain (GLS) [51,52] or Cardiac MRI might have detected subtler changes in myocardial mechanics.

## 5. Conclusions

In this real-world cohort of patients with acute coronary syndrome treated with PCI, the initiation of SGLT2 inhibitors at index hospitalization was associated with a distinct high-risk clinical phenotype characterized by diabetes, lower ejection fraction, and significant myocardial hypertrophy. While these patients exhibited favorable unadjusted trends in reverse remodeling—particularly regression of LV mass—multivariable analysis revealed that these trajectories were primarily driven by the severity of the baseline substrate rather than the independent effect of the pharmacotherapy. Critically, despite their significantly higher cardiometabolic risk burden, SGLT2-treated patients achieved structural outcomes comparable to lower-risk controls, suggesting that early SGLT2 inhibitors have been associated with stabilization of the adverse remodeling expected in this high-risk phenotype, effectively aligning their trajectory with that of a lower-risk population. These findings do not diminish the established prognostic role of SGLT2 inhibitors in heart failure but highlight the phenotype-dependent nature of structural benefits in the complex, multifactorial setting of acute post-infarction care. Future research should leverage advanced imaging and uniform follow-up to further delineate the specific responders to this therapy in the post-ACS landscape.

## Figures and Tables

**Table 1:** Baseline clinical characteristics by treatment group.

Characteristic	SGLT2i Group (n = 71)	Non-SGLT2i Group (n = 167)	*p*-Value
Age (years), mean ± SD	61.5 ± 10.2	59.8 ± 10.8	0.277
Male sex, n (%)	55 (77.5%)	126 (75.4%)	0.867
Diabetes mellitus, n (%)	40 (56.3%)	43 (25.7%)	<0.001
Hypertension, n (%)	62 (87.3%)	143 (85.6%)	0.888
Chronic kidney disease, n (%)	10 (14.1%)	10 (6.0%)	0.071
Type of ACS (STEMI), n (%)	60 (84.5%)	146 (87.4%)	0.55
Time to follow-up (days), mean ± SD	290.8 ± 208.1	284.5 ± 243.8	0.848
ACEi/ARB/ARNi, n (%)	56 (78.9%)	125 (74.9%)	0.51
Beta-blockers, n (%)	54 (76.1%)	123 (73.7%)	0.70
MRA, n (%)	63 (88.7%)	131 (78.4%)	0.091
Loop diuretics, n (%)	58 (81.7%)	126 (75.4%)	0.377

Abbreviations: ACEi, angiotensin-converting enzyme inhibitor; ACS, acute coronary syndrome; ARB, angiotensin receptor blocker; ARNi, angiotensin receptor-neprilysin inhibitor; MRA, mineralocorticoid receptor antagonist; SD, standard deviation; STEMI, ST-segment elevation myocardial infarction, ±, standard deviation.

**Table 2:** Baseline echocardiographic parameters by treatment group.

Parameter	SGLT2i Group (n = 71)	Non-SGLT2i Group (n = 167)	*p*-Value
LVEF (%), mean ± SD	38.3 ± 6.5	43.3 ± 7.8	<0.001
LVM (g), mean ± SD	249.8 ± 69.3	218.3 ± 60.0	<0.001
LVMi (g/m^2^), mean ± SD	125.8 ± 33.7	110.3 ± 29.1	<0.001
LVEDV (mL), mean ± SD	126.4 ± 41.9	111.6 ± 31.2	0.013
RWT, mean ± SD	0.48 ± 0.08	0.49 ± 0.08	0.33

Abbreviations: LVEDV, left ventricular end-diastolic volume; LVEF, left ventricular ejection fraction; LVM, left ventricular mass; LVMi, indexed left ventricular mass; RWT, relative wall thickness; SD, standard deviation.

**Table 3:** Multivariable linear regression results for echocardiographic outcomes.

Outcome	Predictor	β Coefficient (95% CI)	*p*-Value
ΔLVEF (%)	SGLT2i Use	+1.68 (−0.27 to 3.64)	0.091
ΔLVM (g)	SGLT2i Use	+2.36 (−10.60 to 15.31)	0.72
ΔLVMi (g/m^2^)	SGLT2i Use	−1.76 (−8.30 to 4.77)	0.60
ΔRWT	SGLT2i Use	+0.016 (−0.004 to 0.037)	0.113

Models adjusted for baseline value of the outcome variable and time interval between baseline and follow-up echocardiography. Abbreviations: CI, confidence interval; LVEF, left ventricular ejection fraction; LVM, left ventricular mass; LVMi, indexed left ventricular mass; RWT, relative wall thickness; SGLT2i, sodium-glucose cotransporter-2 inhibitor.

**Table 4:** Multivariable Linear Regression Models with SGLT2i × Diabetes Interaction Terms.

**Outcome**	**Predictor**	**β Coefficient**	**95% CI**	***p*** **-Value**
ΔLVEF (%)	SGLT2	+1.607	(−1.11, 4.33)	0.246
	Diabet	+1.176	(−1.17, 3.53)	0.325
	SGLT2_x_Diabet	−0.481	(−4.43, 3.46)	0.810
R^2^ = 0.165				
ΔLVMi (g/m^2^)	SGLT2	−0.580	(−9.56, 8.40)	0.899
	Diabet	−1.063	(−9.00, 6.88)	0.792
	SGLT2_x_Diabet	−1.622	(−14.97, 11.73)	0.811
R^2^ = 0.355				

## Data Availability

The data presented in this study are available on request from the corresponding author. The data are not publicly available due to privacy restrictions.

## Abbreviations

The following abbreviations are used in this manuscript:

AMI	Acute Myocardial Infarction
BSA	Body Surface Area
ESC	European Society of Cardiology
HF	Heart Failure
HFrEF	Heart Failure with Reduced Ejection Fraction
IQR	Interquartile Range
LV	Left Ventricular
LVEDD	Left Ventricular End-Diastolic Diameter
LVEF	Left Ventricular Ejection Fraction
LVM	Left Ventricular Mass
LVMi	Left Ventricular Mass Index
NHE1	Sodium-Hydrogen Exchanger 1
NSTE-ACS	Non-ST-Segment Elevation Acute Coronary Syndrome
PCI	Percutaneous Coronary Intervention
RWT	Relative Wall Thickness
SD	Standard Deviation
SGLT2i	Sodium-Glucose Cotransporter 2 Inhibitor
STEMI	ST-Elevation Myocardial Infarction
TTE	Transthoracic Echocardiogram

## References

[1] Salari N., Morddarvanjoghi F., Abdolmaleki A., Rasoulpoor S., Khaleghi A.A., Hezarkhani L.A., Shohaimi S., Mohammadi M. (2023). The global prevalence of myocardial infarction: A systematic review and meta-analysis. BMC Cardiovasc Disord..

[2] Rathore V., Singh N., Mahat R.K. (2018). Risk Factors of Acute Myocardial Infarction: A Review. Eurasian J. Med. Investig..

[3] Bagai A., Dangas G.D., Stone G.W., Granger C.B. (2014). Reperfusion Strategies in Acute Coronary Syndromes. Circ Res..

[4] Armstrong P.W., Bogaerts K., Welsh R., Sinnaeve P.R., Goldstein P., Pages A., Danays T., Van de Werf F. (2020). The Second Strategic Reperfusion Early After Myocardial Infarction (STREAM-2) study optimizing pharmacoinvasive reperfusion strategy in older ST-elevation myocardial infarction patients. Am. Heart J..

[5] Calvieri C., Riva A., Sturla F., Dominici L., Conia L., Gaudio C., Miraldi F., Secchi F., Galea N. (2023). Left Ventricular Adverse Remodeling in Ischemic Heart Disease: Emerging Cardiac Magnetic Resonance Imaging Biomarkers. J. Clin. Med..

[6] Pfeffer M.A., Braunwald E. (1990). Ventricular remodeling after myocardial infarction. Experimental observations and clinical implications. Circulation.

[7] Sutton M.G.S.J., Sharpe N. (2000). Left Ventricular Remodeling After Myocardial Infarction: Pathophysiology and Therapy. Circulation.

[8] Yin X., Yin X., Pan X., Zhang J., Fan X., Li J., Zhai X., Jiang L., Hao P., Wang J. (2023). Post-myocardial infarction fibrosis: Pathophysiology, examination, and intervention. Front. Pharmacol..

[9] Sanjaya A. (2025). Transcriptomic dynamics of cardiac remodeling after myocardial infarction. Sci Rep..

[10] Marcos-Garcés V., Bertolín-Boronat C., Merenciano-González H., Mas M.L.M., Alberola J.I.C., López-Bueno L., Rubio A.P., Pérez-Solé N., Ríos-Navarro C., de Dios E. (2025). Left Ventricular Remodeling After Myocardial Infarction—Pathophysiology, Diagnostic Approach and Management During Cardiac Rehabilitation. Int. J. Mol. Sci..

[11] Frangogiannis N.G. (2014). The inflammatory response in myocardial injury, repair, and remodelling. Nat. Rev. Cardiol..

[12] Leo I., Salerno N., Figliozzi S., Cersosimo A., Ielapi J., Stankowski K., Bisaccia G., Dellegrottaglie S., Canino G., De Rosa S. (2025). Effect of SGLT2 inhibitors on cardiac structure and function assessed by cardiac magnetic resonance: A systematic review and meta-analysis. Cardiovasc. Diabetol..

[13] Wu X., Wu M., Huang H., Liu Z., Huang H., Wang L. (2025). A predictive model for heart failure with preserved ejection fraction following acute myocardial infarction: The HFpEF-AMI score. BMC Cardiovasc. Disord..

[14] Liang J., Zhang Z. (2023). Predictors of in-hospital heart failure in patients with acute anterior wall ST-segment elevation myocardial infarction. Int. J. Cardiol..

[15] Wichterle D. (2010). Risk Stratification in Post-myocardial Infarction Patients. Eur. Cardiol. Rev..

[16] Hellermann J.P., Jacobsen S.J., Gersh B.J., Rodeheffer R.J., Reeder G.S., Roger V.L. (2002). Heart failure after myocardial infarction: A review. Am. J. Med..

[17] Sekaran N.K., Crowley A.L., de Souza F.R., Resende E.S., Rao S.V. (2017). The Role for Cardiovascular Remodeling in Cardiovascular Outcomes. Curr. Atheroscler. Rep..

[18] Greene S.J., Mentz R.J., Fiuzat M., Butler J., Solomon S.D., Ambrosy A.P., Mehta C., Teerlink J.R., Zannad F., O’Connor C.M. (2018). Reassessing the Role of Surrogate End Points in Drug Development for Heart Failure. Circulation.

[19] Brener M.I., Kapadia S.R., Burkhoff D. (2023). Reverse Left Ventricular Remodeling with Transcatheter Interventions in Chronic Heart Failure Syndromes: An Updated Appraisal of the Device Landscape. J. Soc. Cardiovasc. Angiogr Interv..

[20] Leancă S.A., Crișu D., Petriș A.O., Afrăsânie I., Genes A., Costache A.D., Tesloianu D.N., Costache I.I. (2022). Left Ventricular Remodeling after Myocardial Infarction: From Physiopathology to Treatment. Life.

[21] Mateoc T., Dumitrascu A.-L., Flangea C., Puscasiu D., Vlad T., Popescu R., Marina C., Vlad D.-C. (2025). SGLT2 Inhibitors: From Structure–Effect Relationship to Pharmacological Response. Int. J. Mol. Sci..

[22] McMurray J.J.V., Solomon S.D., Inzucchi S.E., Køber L., Kosiborod M.N., Martinez F.A., Ponikowski P., Sabatine M.S., Anand I.S., Bělohlávek J. (2019). Dapagliflozin in Patients with Heart Failure and Reduced Ejection Fraction. N. Engl. J. Med..

[23] Packer M., Anker S.D., Butler J., Filippatos G., Pocock S.J., Carson P., Januzzi J., Verma S., Tsutsui H., Brueckmann M. (2020). Cardiovascular and Renal Outcomes with Empagliflozin in Heart Failure. N. Engl. J. Med..

[24] Anker S.D., Butler J., Filippatos G., Ferreira J.P., Bocchi E., Böhm M., Brunner–La Rocca H.-P., Choi D.-J., Chopra V., Chuquiure-Valenzuela E. (2021). Empagliflozin in Heart Failure with a Preserved Ejection Fraction. N. Engl. J. Med..

[25] Verma S., McMurray J.J.V. (2018). SGLT2 inhibitors and mechanisms of cardiovascular benefit: A state-of-the-art review. Diabetologia.

[26] Vahid S.S., Jahromi M.S., Moukarbel G.V., Hill J.W. (2025). Mechanistic insights and clinical horizons of SGLT2 inhibitors in heart failure management. Trends Cardiovasc. Med..

[27] Suciu I.M., Luca S.A., Crișan S., Cozlac A.-R., Stoica S., Luca C.T., Timar B., Gaita D. (2025). Do SGLT2 Inhibitors Improve Cardiovascular Outcomes After Acute Coronary Syndrome Regardless of Diabetes? A Systematic Review and Meta-Analysis. Medicina.

[28] Harrington J., Udell J.A., Jones W.S., Anker S.D., Bhatt D.L., Petrie M.C., Vedin O., Sumin M., Zwiener I., Hernandez A.F. (2022). Empagliflozin in patients post myocardial infarction rationale and design of the EMPACT-MI trial. Am. Heart J..

[29] James S., Erlinge D., Storey R.F., McGuire D.K., de Belder M., Eriksson N., Andersen K., Austin D., Arefalk G., Carrick D. (2024). Dapagliflozin in Myocardial Infarction without Diabetes or Heart Failure. NEJM Evid..

[30] Butler J., Jones W.S., Udell J.A., Anker S.D., Petrie M.C., Harrington J., Mattheus M., Zwiener I., Amir O., Bahit M.C. (2024). Empagliflozin after Acute Myocardial Infarction. N. Engl. J. Med..

[31] Prabhu S.D., Frangogiannis N.G. (2016). The Biological Basis for Cardiac Repair After Myocardial Infarction: From Inflammation to Fibrosis. Circ. Res..

[32] Das J., Sah A.K., Choudhary R.K., Elshaikh R.H., Bhui U., Chowdhury S., Abbas A.M., Shalabi M.G., Siddique N.A., Alshammari R.R. (2025). Network Pharmacology Approaches to Myocardial Infarction Reperfusion Injury: Exploring Mechanisms, Pathophysiology, and Novel Therapies. Biomedicines.

[33] Von Elm E., Altman D.G., Egger M., Pocock S.J., Gøtzsche P.C., Vandenbroucke J.P. (2008). The Strengthening the Reporting of Observational Studies in Epidemiology (STROBE) statement: Guidelines for reporting observational studies. J. Clin. Epidemiol..

[34] Manolis A.A., Manolis T.A., Manolis A.S. (2023). Neurohumoral Activation in Heart Failure. Int. J. Mol. Sci..

[35] Rosano G., Vitale C. (2018). Metabolic Modulation of Cardiac Metabolism in Heart Failure. Card. Fail. Rev..

[36] Rosano G.M., Vitale C., Spoletini I. (2015). Metabolic approach to heart failure: The role of metabolic modulators. Egypt Heart. J..

[37] Taha M., Lopaschuk G.D. (2007). Alterations in energy metabolism in cardiomyopathies. Ann. Med..

[38] Peterzan M.A., Lygate C.A., Neubauer S., Rider O.J. (2017). Metabolic remodeling in hypertrophied and failing myocardium: A review. Am. J. Physiol. Heart Circ. Physiol..

[39] Zhang J., Abel E.D. (2018). Effective Metabolic Approaches for the Energy Starved Failing Heart: Bioenergetic Resiliency via Redundancy or Something Else?. Circ. Res..

[40] Paraskevaidis I., Kourek C., Farmakis D., Tsougos E. (2024). Heart Failure: A Deficiency of Energy—A Path Yet to Discover and Walk. Biomedicines.

[41] Lopaschuk G.D., Verma S. (2020). Mechanisms of Cardiovascular Benefits of Sodium Glucose Co-Transporter 2 (SGLT2) Inhibitors. JACC Basic Transl. Sci..

[42] Saucedo-Orozco H., Voorrips S.N., Yurista S.R., de Boer R.A., Westenbrink B.D. (2022). SGLT2 Inhibitors and Ketone Metabolism in Heart Failure. J. Lipid Atheroscler..

[43] Manolis A.S., Manolis T.A., Manolis A.A. (2023). Ketone Bodies and Cardiovascular Disease: An Alternate Fuel Source to the Rescue. Int. J. Mol. Sci..

[44] Purushothaman S., Nair R.R., Harikrishnan V.S., Fernandez A.C. (2011). Temporal relation of cardiac hypertrophy, oxidative stress, and fatty acid metabolism in spontaneously hypertensive rat. Mol. Cell Biochem..

[45] Ma R., Lu D., Yang Z., Ji X., Tian R., Xu F., Chen Y., Li C. (2025). Emerging therapy strategies for energy metabolism in acute myocardial infarction. J. Transl. Med..

[46] Karwi Q.G., Biswas D., Pulinilkunnil T., Lopaschuk G.D. (2020). Myocardial Ketones Metabolism in Heart Failure. J. Card. Fail..

[47] Jensch P.-J., Stiermaier T., Reinstadler S.J., Feistritzer H.-J., Desch S., Fuernau G., de Waha-Thiele S., Thiele H., Eitel I. (2022). Prognostic relevance of peri-infarct zone measured by cardiovascular magnetic resonance in patients with ST-segment elevation myocardial infarction. Int. J. Cardiol..

[48] Matteucci A., Pandozi C., Bonanni M., Mariani M.V., Sgarra L., Nesti L., Pierucci N., La Fazia V.M., Lavalle C., Nardi F. (2025). Impact of empagliflozin and dapagliflozin on sudden cardiac death: A systematic review and meta-analysis of adjudicated randomized evidence. Heart Rhythm..

[49] Verma S., Mazer C.D., Yan A.T., Mason T., Garg V., Teoh H., Zuo F., Quan A., Farkouh M.E., Fitchett D.H. (2019). Effect of Empagliflozin on Left Ventricular Mass in Patients with Type 2 Diabetes Mellitus and Coronary Artery Disease: The EMPA-HEART CardioLink-6 Randomized Clinical Trial. Circulation.

[50] Dyck J.R., Sossalla S., Hamdani N., Coronel R., Weber N.C., Light P.E., Zuurbier C.J. (2022). Cardiac mechanisms of the beneficial effects of SGLT2 inhibitors in heart failure: Evidence for potential off-target effects. J. Mol. Cell. Cardiol..

[51] Taub C.C., Stainback R.F., Abraham T., Forsha D., Garcia-Sayan E., Hill J.C., Hung J., Mitchell C., Rigolin V.H., Sachdev V. (2025). Guidelines for the Standardization of Adult Echocardiography Reporting: Recommendations from the American Society of Echocardiography. J. Am. Soc. Echocardiogr..

[52] Smiseth O.A., Rider O., Cvijic M., Valkovič L., Remme E.W., Voigt J.-U. (2025). Myocardial Strain Imaging. JACC Cardiovasc Imaging.

